# A Comprehensive Examination of Changes in Psychological Flexibility Following Acceptance and Commitment Therapy for Chronic Pain

**DOI:** 10.1007/s10879-016-9328-5

**Published:** 2016-03-02

**Authors:** Whitney Scott, Katie E. J. Hann, Lance M. McCracken

**Affiliations:** Health Psychology Section, Psychology Department, Institute of Psychiatry, Psychology & Neuroscience, King’s College London, Guy’s Campus, 5th Floor Bermondsey Wing, London, SE1 9RT UK; INPUT Pain Management, Guy’s and St Thomas’ NHS Foundation Trust, London, UK

**Keywords:** Chronic pain, Psychological flexibility, Acceptance and commitment therapy

## Abstract

Acceptance and commitment therapy (ACT) for chronic pain aims to improve patient functioning by fostering greater psychological flexibility. While promising, ACT treatment process research in the context of chronic pain so far has only focused on a few of the processes of psychological flexibility. Therefore, this study aimed to more comprehensively examine changes in processes of psychological flexibility following an ACT-based treatment for chronic pain, and to examine change in these processes in relation to improvements in patient functioning. Individuals with chronic pain attending an interdisciplinary ACT-based rehabilitation program completed measures of pain, functioning, depression, pain acceptance, cognitive fusion, decentering, and committed action at pre- and post-treatment and during a nine-month follow-up. Significant improvements were observed from pre- to post-treatment and pre-treatment to follow-up on each of the treatment outcome and process variables. Regression analyses indicated that change in psychological flexibility processes cumulatively explained 6–27 % of the variance in changes in functioning and depression over both assessment periods, even after controlling for changes in pain intensity. Further research is needed to maximize the effectiveness of ACT for chronic pain, and to determine whether larger improvements in the processes of psychological flexibility under study will produce better patient outcomes, as predicted by the psychological flexibility model.

## Introduction

The efficacy of cognitive behavioral therapy (CBT) for chronic pain is now well established (Williams et al. 2012). At the same time, CBT continues to develop. In recent years there has been growing interest in acceptance and commitment therapy (ACT), a newer form of CBT, for improving chronic pain outcomes. The focus within ACT is to help people disengage from unsuccessful efforts to control or avoid pain, and instead move toward pursuing goals and values more consistently (McCracken 2005; Dahl and Lundgren 2006). The efficacy and effectiveness of ACT for chronic pain is supported by 11 randomized controlled trials and numerous uncontrolled trials (Veehof et al. 2011; Hann and McCracken 2014).

One particular advantage of ACT is its explicit connection with a guiding theoretical model. Theoretically, ACT is based on the psychological flexibility model, a model of human behavior that applies a functional, contextual and, above all, pragmatic viewpoint (Hayes et al. 2006, 2013; McCracken and Morley 2014). Psychological flexibility has been described as the capacity to persist with or change behavior in a manner that incorporates conscious and open contact with thoughts, feelings, and sensory experiences, and in a manner that reflects one’s values and goals (McCracken and Morley 2014). Six processes are suggested to underlie psychological flexibility: acceptance, cognitive defusion, flexible present-focused attention, self-as-context, values-based action, and committed action. Briefly, these processes reflect openness to experience as opposed to avoidance, moment-to-moment awareness of experiences and perspective taking rather than being disconnected from the present or entangled in psychological experiences, and an active focus on values and goals rather than on problems. While these facets are described as distinct to a degree, it is recognized that they share overlap in some of the psychological qualities they reflect (Hayes et al. 2011). Conversely, psychological inflexibility includes typically dominant avoidance-promoting influences, usually associated with thoughts and feelings, which restrict behavioral choice and coordinate behavior that is inconsistent with an individual’s goals or values. Each facet of psychological flexibility has a corresponding facet in psychological inflexibility (Hayes et al. 2006). The processes that comprise psychological flexibility are rooted in basic science research that defines thinking and cognitive processes in terms of principles of operant conditioning (Barnes-Holmes and Barnes-Holmes 2000; Barnes-Holmes et al. 2000; Dymond et al. 2010).

Most studies of ACT-based treatment for pain have measured processes of psychological flexibility to some extent. A number of studies have reported that ACT is associated with significant and meaningful changes in components of psychological flexibility, including acceptance, values-based action, and mindfulness. In turn, as predicted by the model, improvements in these process measures have been associated with improvements in measures of daily functioning (McCracken and Gutiérrez-Martínez 2011; Wicksell et al. 2013; Vowles et al. 2011; Wicksell et al. 2010; Vowles et al. 2014b).

While promising, research on psychological flexibility in relation to treatment for chronic pain so far has largely neglected the facets of cognitive defusion, self-as-context, and committed action. This research has been hampered by a lack of validated measures of these processes in people with pain. Recently, however, measures of cognitive fusion/defusion and committed action have been developed and validated. As predicted, preliminary data indicate these measures are associated with emotional well-being and general daily functioning in cross-sectional analyses (McCracken 2013; McCracken et al. 2013; Trompetter et al. 2013). Although not a comprehensive measure of self-as-context, a measure of decentering which contains items tapping cognitive defusion and self-as-context, was also recently validated and shown to be associated with better patient functioning in one cross-sectional study (McCracken et al. 2014b). To date, no study has examined change in these processes during ACT-based treatment for pain.

Thus, with one exception (Vowles et al. 2014b), ACT treatment process research has not yet focused widely on all of the key facets of psychological flexibility. More comprehensive assessment methods are needed to examine the contributions of change in a wider range of these processes to changes in chronic pain outcomes. Given theoretical and empirical overlap in these processes and their corresponding measurement tools, investigation of the shared and unique associations between these processes and treatment outcomes is needed to determine their individual specificity and incremental utility. A more integrative examination of change in these processes may inform treatment refinements that may ultimately improve treatment outcomes.

The purpose of this study was to investigate the relative magnitude of changes in pain acceptance, cognitive fusion, decentering, and committed action following ACT-based treatment for pain. Relatedly, the study sought to identify the shared and unique associations between changes in these treatment process variables and changes in daily functioning. People with chronic pain attending an interdisciplinary ACT-based rehabilitation program completed measures of pain, daily functioning, depression, and processes of psychological flexibility at pre- and post-treatment and at a nine-month follow-up. It was predicted that each of the psychological flexibility process measures would show significant improvements from pre- to post-treatment and to follow-up. It was also predicted that improvements in each of these process measures would uniquely contribute to improvements in daily functioning.

## Methods

### Participants

This was an observational cohort study during which data were collected in the course of routine clinical assessment and treatment delivery procedures. Participants for this study were consecutive referrals to a four-week, residential, interdisciplinary pain management program in London, UK, who began treatment between January 2012 and October 2013. Participants were selected if they had pain of greater than 3 months duration, significant pain-related distress and disability, and were judged as likely to benefit from the program based on assessment by a specialist physiotherapist and psychologist.

Initially, 473 individuals began the treatment program. Of these, 13 did not consent to have their data used for research purposes, another 34 voluntarily discontinued treatment, and 42 had missing data on one or more clinical outcome or treatment process variable. Therefore, the sample of participants with complete data for this study at pre- and post-treatment was 384. For the pre- to follow-up analyses, an additional 170 individuals did not provide data for the follow-up assessment. Therefore, the sample providing data for the pre- to nine-month follow-up period was 214.

Table 1 displays demographic information of the sample with complete pre- and post-treatment data (n = 384). The average age of the sample was 46.4 years (SD = 11.6). Most of the participants were women (66.4 %), white British (72.1 %), with an average of 13.2 years of education (SD = 3.9). Over half (50.8 %) of the sample was unemployed at the start of treatment due to pain. The majority of participants lived with a partner (49.6 %). The median duration of pain was 99.0 months (range 3–704 months). The most commonly reported primary area of pain was the lower back (42.7 %).

**Table 1:** Participant demographics (*n* = 384)

	Mean (SD) or *n* (%)
Age at assessment	46.4 (11.6)
Pain duration (months)	99.0 (3–704)^a^
Years education	13.2 (3.9)
Sex	
Male	129 (33.6 %)
Female	255 (66.4 %)
Primary pain site	
Head, face or mouth	11 (2.9 %)
Neck region	32 (8.3 %)
Upper shoulder/limbs	35 (9.1 %)
Chest region	5 (1.3 %)
Abdominal region	6 (1.6 %)
Lower back/spine	164 (42.7 %)
Lower limbs	58 (15.1 %)
Pelvic region	4 (1.0 %)
Anal/genital	4 (1.0 %)
Generalized	65 (16.9 %)
Ethnicity	
Black	62 (16.2 %)
White	277 (72.1 %)
Asian	27 (7.0 %)
Mixed	18 (4.7 %)
Living status	
Alone	94 (24.5 %)
With partner and/or children	248 (64.6 %)
With other family members	32 (8.3 %)
With friends/flatmates	9 (2.3 %)
Missing	1 (0.3 %)
Work status	
Employed	111 (28.9 %)
Unemployed due to pain	195 (50.8 %)
Unemployed for other reason	12 (3.1 %)
Other (retired, homemaker, student, etc.)	66 (17.2 %)

^a^Pain duration showed a skewed distribution and is thus reported in terms of the median and range

### Procedure

On the first day of the treatment course patients were asked to complete standard baseline assessment material. Patients completed self-report measures of pain intensity, physical and social functioning, symptoms of depression, and measures of psychological flexibility processes. The outcome measures described below are consistent with the IMMPACT recommendations regarding important outcome measures for treatments for people with chronic pain (Dworkin et al. 2005). During this assessment, patients also provided background information, including their sex, age, ethnicity, pain location and duration, living situation, years of education, and work status. Patients completed the same self-report measures again at the completion of treatment and during a nine-month follow-up appointment. All patients provided written informed consent to have their data used for the purpose of research. The research database and study were granted local ethics and NHS R&D approvals.

### Pre- and Post-treatment and Follow-Up Assessment Measures

#### Pain Intensity

Participants rated their average pain in the past week on a standard scale from 0 (no pain) to 10 (extremely intense pain).

#### Daily Functioning

The SF-36 (Ware and Sherbourne 1992) is a standardized 36-item measure of health status. The SF-36 yields eight subscale scores assessing various domains of life functioning. Of the eight subscales, only the physical functioning and social functioning subscales were used for the purpose of the present study. Higher scores on these subscales indicate better function in these domains. In the current study, the physical functioning subscale demonstrated good internal consistency (Chronbach’s α = 0.85); the social functioning subscale showed poor internal consistency (Chronbach’s α = 0.58). However, the social functioning subscale of the SF-36 has shown good reliability (Chronbach’s α > 0.80) in previous studies and is frequently used among samples of people with pain (Bergman et al. 2004; Elliott et al. 2003; Wittink et al. 2004).

#### Depression

The PHQ-9 (Kroenke et al. 2001) was used to measure the severity of depression symptoms based on the standard DSM-IV diagnostic criteria. On this measure, people are asked to report on the frequency with which they experience nine symptoms of depression from 0 (not at all) to 3 (nearly every day). Higher scores on the PHQ-9 indicate more severe symptoms. The PHQ-9 has been well validated and shown to sensitively discriminate between people with and without diagnoses of Major Depression in people with chronic pain (Choi et al. 2014). In the current sample, the PHQ-9 achieved good internal reliability (Chronbach’s α = 0.83).

#### Chronic Pain Acceptance

Chronic pain acceptance is a process of engagement in activities that include pain and the cessation of unsuccessful efforts to control pain so that important life activities may be pursued. The Chronic Pain Acceptance Questionnaire (CPAQ) was used to measure pain acceptance (McCracken et al. 2004). On the CPAQ, people rate 20-items on a seven point scale ranging from 0 (never true) to 6 (always true). The CPAQ includes items such as, “I am getting on with the business of living no matter what my level of pain is”. Higher scores indicate greater acceptance on this measure. A systematic review of questionnaires assessing acceptance of chronic pain concluded that the CPAQ demonstrates the highest performance in terms of its psychometric properties relative to other measures of pain acceptance (Reneman et al. 2010). The CPAQ showed good internal reliability in the present sample (Chronbach’s α = 0.85). In a previous study, the test re-test reliability of the CPAQ following an average waitlist interval of approximately 4 months was *r* = 0.75 (McCracken and Eccleston 2005).

#### Cognitive Fusion

Cognitive fusion includes the excessive influence of thoughts on experience and action, and an inability to experience a distinction between thoughts and the situations, events, or people to which they refer (Hayes et al. 2006). The 13-item cognitive fusion questionnaire was used to measure cognitive fusion (Gillanders et al. 2014). On this measure, participants rate items on a 7-point scale from 1 (never true) to 7 (always true). An example item from the CFQ is, “My thoughts cause me distress or emotional pain”. Higher scores on this measure reflect greater cognitive fusion. The CFQ has previously demonstrated good reliability (Chronbach’s α = 0.87) in a sample of people with chronic pain, and uniquely predicted patient functioning and mental health even after controlling for chronic pain acceptance (McCracken et al. 2013). The CFQ demonstrated good internal reliability in the present sample (Chronbach’s α = 0.85).

#### Decentering

The Experiences Questionnaire (EQ) was used to measure decentering (Fresco et al. 2007). The EQ contains a 14-item decentering subscale and a six-item rumination subscale. Decentering reflects the ability to observe one’s thoughts and feelings as temporary events in the mind, rather than as ‘true’ reflections of the self or one’s circumstances (Safran and Segal 1996). In contrast, rumination reflects a repetitive cycling of thought about reasons for one’s emotional state. On this measure, people rate each item on a 5-point scale ranging from 1 (never) to 5 (all the time). Rumination items are reverse scored and the total EQ score is calculated as the sum of decentering and rumination subscale scores. An example item from the EQ is, “I view things from a wider perspective”. Higher scores on the EQ indicate greater decentering. The EQ has previously been shown to have good reliability (Chronbach’s α = 0.86 and 0.72 for the decentering and rumination subscales, respectively), to be significantly correlated with measures of functioning and mental health, and to uniquely predict patient outcomes in people with chronic pain even when controlling for chronic pain acceptance (McCracken et al. 2014b). The EQ had good internal reliability in the current study (Chronbach’s α = 0.81).

#### Committed Action

Committed action includes flexible persistence in goal-directed behavior (Hayes et al. 2006). Committed action was assessed with the 18-item version of the committed action questionnaire (McCracken 2013). The measure includes positively and negatively phrased items. An example item from the CAQ is, “When a goal is difficult to reach, I am able to take small steps to reach it”. Respondents are asked to rate the extent to which each of the items applies to them on a 7-point scale ranging from ‘never true’ to ‘always true’. Negatively phrased items are reversed prior to computing a total score on this scale. Higher total scores on the CAQ thus reflect greater committed action. In a previous study, the CAQ showed excellent internal reliability (Chronbach’s α = 0.91), and uniquely predicted functioning and mental health outcomes even when controlling for chronic pain acceptance in a sample of people with chronic pain (McCracken et al. 2014a). The CAQ showed good internal reliability in the present sample (Chronbach’s α = 0.88).

### Treatment Program

The treatment used principles and methods of ACT within an interdisciplinary rehabilitation context. The goal of treatment is to improve overall patient functioning. Treatment was provided in a group format and consisted of four full days of treatment per week for 4 weeks. Treatment was delivered by a team of psychologists, occupational and physical therapists, nurses, and physicians. The methods were designed to explicitly target the processes of psychological flexibility: Openness to experiencing pain and unwanted emotions; defusion from the content of thoughts; the ability to flexibly focus attention on the present moment; the ability to adopt the perspective of an observer of physical sensations, thoughts, and feelings, and to experience these as separate from oneself; and, to take values-based and committed actions. To this end, experiential exercises, metaphors, mindfulness practice, cognitive defusion techniques, values clarification, goal-setting, and behavioral activation methods were used (McCracken 2005; Hayes and Smith 2005; Dahl et al. 2005).

### Data Analysis

Means and standard deviations were computed for pre- and post-treatment and follow-up assessment measures. Independent samples *t*-tests were computed to examine differences on assessment variables for treatment completers and non-completers and patients with and without follow-up data. Given different samples sizes across the assessment points, separate repeated-measures one-way analyses of variance (ANOVAs) were computed to determine the statistical significance of changes in clinical outcome and treatment process variables for the pre- to post-treatment and pre-treatment to follow-up assessments. Within-subjects effect sizes (Cohen’s d) were computed as the difference between pre- and post-treatment or follow-up means divided by the pooled standard deviation. Consistent with Cohen’s guidelines, effect sizes were interpreted as small (>0.20), medium (>0.50), or large (>0.80) (Cohen 1988).

Pearson correlations were computed to examine the associations among change in clinical outcome and treatment process variables for both the pre- to post-treatment and pre-treatment to follow-up periods. For these analyses, residualized change scores were first computed for all variables. For each variable, the baseline value was used to predict the post-treatment or follow-up value of the variable in a regression analysis, and the residualized change score was computed as the difference between the post-treatment or follow-up score with the baseline score covaried out. Pearson correlations were then computed to examine the associations between residualized change scores on assessment variables and change ratings.

A series of hierarchical multiple regression analyses were computed to examine the shared and unique contributions of change in treatment process variables to change in each of the clinical outcomes for the pre- to post-treatment and pre- to follow-up periods. For the analysis predicting change in pain intensity, changes in pain acceptance, cognitive fusion, decentering, and committed action were simultaneously entered in one step. For the analyses predicting changes in physical and social functioning and depression, changes in pain were entered in the first step as a control variable and the four process variables were entered in the second step. Given potential for high inter-correlations between process measures, simultaneous entry of these variables enables examination of their shared and unique associations with treatment outcomes by examining the magnitude of the R^2^ change value for the step and the individual beta weights from the final regression equation, respectively. For each of the regression analyses, the tolerance and variance inflation factor indices were within acceptable limits indicating no problems with multicollinearity.

## Results

### Preliminary Analyses

Independent samples t-tests were computed to investigate differences between participants who completed treatment and those who did not. Treatment completers scored significantly higher on the social functioning subscale of the SF-36 at the beginning of treatment (M = 34.91; SD = 22.89) than those who did not complete treatment (M = 24.63; SD = 16.71), *t* (457) = 2.56, *p* = 0.01. No differences were observed between treatment completers and non-completers on any other clinical outcome or treatment process variable.

T-tests were likewise computed to compare post-treatment scores for participants who provided nine-month follow-up data and those who did not. Participants with and without follow-up data showed significantly different scores on a number of post-treatment outcome and process variables: pain acceptance (Follow-up: *M* = 62.69, SD = 18.37; No follow-up, *M* = 58.34, SD = 19.73), *t* (382) = 2.23, *p* < 0.05; cognitive fusion (Follow-up: *M* = 49.35, SD = 14.56; No follow-up, *M* = 52.38, SD = 15.93), *t* (382) = −1.94, *p* = 0.05; decentering (Follow-up: *M* = 61.36, SD = 10.14; No follow-up, *M* = 58.77, SD = 11.17), *t* (382) = 2.38, *p* < 0.05; depression (follow-up: *M* = 10.61, SD = 6.27; No follow-up, *M* = 12.81, SD = 6.28), *t* (382) = −3.42, *p* = 0.001; and social functioning (follow-up: *M* = 56.19, SD = 25.11; No follow-up, *M* = 50.22, SD = 24.27), *t* (382) = 2.35, *p* < 0.05).

### Post-treatment and Follow-Up Outcomes

Table 2 shows mean scores on clinical outcome and treatment process variables at pre-treatment, post-treatment, and nine-month follow-up. Repeated-measures ANOVAs indicated significant differences on all study variables between pre- and post-treatment, all *p* values ≤0.05. A large effect was found for pre to post-treatment improvements in depression. Medium effect sizes were seen for pain intensity, physical and social functioning, and chronic pain acceptance. Small effect sizes were observed for committed action and decentering (Table 2). Although statistically significant, the effect size for pre- to post-treatment improvements in cognitive fusion fell below Cohen’s cut-off for a small effect.

**Table 2:** Mean values (standard deviations) and effect sizes for treatment outcome and process variables

Measure	Pre-treatment (*n* = 384)	Post-treatment (*n* = 384)	Follow-up (*n* = 214)	Pre-post *F* (1, 384)	Pre-post *d*	Pre-follow up *F*(1, 214)	Pre-follow up *d*
Pain	7.67 (1.61)	6.55 (1.93)	7.12 (2.00)	141.57**	0.63	11.34*	0.24
SF-36 physical	23.54 (17.76)	35.03 (22.66)	31.64 (23.85)	144.22**	0.56	33.44**	0.35
SF-36 social	34.99 (22.63)	53.55 (24.89)	46.96 (26.97)	200.79**	0.78	29.35**	0.41
PHQ-9	16.69 (6.08)	11.58 (6.36)	13.18 (7.28)	350.95**	0.82	39.65**	0.39
CPAQ	46.46 (18.38)	60.76 (19.08)	60.20 (19.80)	214.72**	0.76	64.93**	0.59
CFQ	52.39 (15.06)	50.69 (15.24)	47.68 (14.85)	7.85*	0.11	19.46**	0.27
EQ	56.79 (10.39)	60.22 (10.67)	60.50 (10.59)	51.73*	0.32	27.04**	0.33
CAQ	59.85 (16.58)	65.43 (16.06)	63.63 (16.74)	69.14**	0.34	11.37*	0.20

*SF-36 physical* physical functioning subscale of SF-36; *SF-36 social* social functioning subscale of SF-36; *PHQ-9* patient health questionnaire, depression module; *CPAQ* chronic pain acceptance questionnaire; *CFQ* cognitive fusion questionnaire; *EQ* experiences questionnaire; *CAQ* committed action questionnaire

* *p* ≤ 0.05, ** *p* ≤ 0.0001

Repeated-measures ANOVAs likewise indicated that all measures showed significant improvements from pre-treatment to the 9-month follow-up assessment, all *p* values ≤0.05. Medium effect sizes were found for improvements in chronic pain acceptance. Small effect sizes were found for pain intensity, physical and social functioning, depression, cognitive fusion, committed action, and decentering (Table 2).

### Treatment Process Analyses

Pearson correlations were computed to examine the contemporaneous associations between changes in treatment process and outcome variables during pre- to post-treatment and pre-treatment to the nine-month follow-up. Residualized change scores for pre- to post-treatment changes and pre-treatment to follow-up changes were used to compute these correlations (Table 2). With the exception of non-significant correlations between changes in cognitive fusion and committed action with changes in pain intensity, changes in all pre- to post-treatment process and outcome variables were significantly inter-correlated in the expected direction. With the exception of a non-significant correlation between changes in decentering and pain intensity, pre-treatment to follow-up changes in all of the treatment process and outcome variables were significantly inter-correlated in the expected direction (Table 3).

**Table 3:** Correlations among pre- to post-treatment and pre-treatment to follow-up change scores for clinical outcome and treatment process variables

	Pain	SF-36 physical	SF-36 social	PHQ	CPAQ	CFQ	EQ
Pre to post (n = 384)							
SF-36 physical	−0.27**						
SF-36 social	−0.17**	0.40**					
PHQ	0.25**	−0.41**	−0.53**				
CPAQ	−0.24**	0.43**	0.46**	−0.45**			
CFQ	0.05	−0.16*	−0.39**	0.46**	−0.43**		
EQ	−0.12*	0.27**	0.35**	−0.44**	0.60**	−0.66**	
CAQ	−0.06	0.27**	0.37**	−0.41**	0.53**	−0.51**	0.48**
Pre to follow-up (n = 214)							
SF-36 physical	−0.26**						
SF-36 Social	−0.34**	0.26**					
PHQ	0.24**	−0.25**	−0.58**				
CPAQ	−0.24**	0.28**	0.45**	−0.41**			
CFQ	0.19*	−0.18*	−0.44*	0.50**	−0.47**		
EQ	−0.13	0.22**	0.38*	−0.42**	0.49**	−0.65**	
CAQ	−0.20*	0.30**	0.44**	−0.46**	0.59**	−0.55**	0.50**

*SF-36 physical* physical functioning subscale of SF-36; *SF-36 social* social functioning subscale of SF-36; *PHQ-9* patient health questionnaire, depression module; *CPAQ* chronic pain acceptance questionnaire; *CFQ* cognitive fusion questionnaire; *EQ* experiences questionnaire; *CAQ* committed action questionnaire

* *p* < 0.05; ** *p* ≤ 0.0001

A series of hierarchical multiple regression analyses were computed to examine the shared and unique contributions of change in each process variable to changes in clinical outcome variables for the pre- to post-treatment and pre- to follow-up periods (Table 4). For the pre- to post-treatment analyses, changes in treatment process variables together accounted for 6–27 % of the variance in changes in clinical outcomes. Change in chronic pain acceptance uniquely predicted change in all of the clinical outcomes. Change in cognitive fusion uniquely predicted changes in social functioning and depression. Change in committed action uniquely predicted change in symptoms of depression. Change in decentering did not uniquely predict changes in any of the clinical outcomes.

**Table 4:** Regression analyses predicting changes in clinical outcomes from changes in treatment process variables for pre- to post-treatment and pre-treatment to 9-month follow-up

	Pre- to post-treatment			Pre-treatment to follow-up		
	∆R^2^	*F* change	β (final)	∆R^2^	*F* change	β (final)
DV: pain						
Step 1	0.06	6.37**		0.07	4.00**	
CPAQ			−0.29**			−0.19*
CFQ			−0.04			−0.11
EQ			0.01			0.07
CAQ			0.08			−0.07
DV: SF-36 physical						
Step 1	0.07	30.38**		0.07	15.88**	
Pain			−0.18**			−0.20**
Step 2	0.15	18.50**		0.08	4.66**	
CPAQ			0.34**			0.10
CFQ			0.08			0.07
EQ			0.06			0.09
CAQ			0.10			0.19*
DV: SF-36 social						
Step 1	0.03	11.92**		0.11	26.90**	
Pain			−0.09			−0.22**
Step 2	0.24	31.42**		0.22	16.79**	
CPAQ			0.32**			0.19*
CFQ			−0.24**			−0.19*
EQ			−0.06			0.06
CAQ			0.09			0.14
DV: PHQ-9						
Step 1	0.06	24.79**		0.06	12.65**	
Pain			0.17**			0.11
Step 2	0.27	38.17**		0.27	20.87**	
CPAQ			−0.18*			−0.10
CFQ			0.24**			0.27**
EQ			−0.08			−0.10
CAQ			−0.15**			−0.19*

*SF-36 physical* physical functioning subscale of SF-36; *SF-36 social* social functioning subscale of SF-36; *PHQ-9* patient health questionnaire, depression module; *CPAQ* chronic pain acceptance questionnaire; *CFQ* cognitive fusion questionnaire; *EQ* experiences questionnaire; *CAQ* committed action questionnaire

* *p* < 0.05, ** *p* ≤ 0.01

For the pre-treatment to follow-up analyses, changes in treatment process variables together accounted for 7–27 % of the variance in changes in clinical outcomes. In contrast to the pre- to post-treatment analyses, change in chronic pain acceptance uniquely predicted only changes in pain intensity and social functioning. Change in committed action uniquely predicted changes in physical functioning and depression. Change in cognitive fusion uniquely predicted changes in social functioning and depression. Change in decentering was not uniquely associated with changes in any of the clinical outcomes.

## Discussion

This study investigated the extent to which different processes of psychological flexibility change following ACT-based treatment for chronic pain and the shared and unique associations between improvements in these processes and improvements in daily functioning. This is the first study in the context of ACT for chronic pain to examine the particular set of processes included: pain acceptance, cognitive fusion, decentering, and committed action.

Consistent with previous research on ACT for pain, significant improvements were observed pre- to post-treatment and at follow-up for pain, physical and social functioning, and depression (Hann and McCracken 2014; Veehof et al. 2011). The magnitudes of these effects were medium and small during the pre- to post-treatment and pre-treatment to 9 months follow-up periods, respectively. Pain acceptance, decentering, and committed action also improved significantly during treatment and these improvements were maintained at follow-up with small to medium effect sizes. Overall, pain acceptance was the process variable showing the greatest improvement and this was maintained through the follow-up period. Interestingly, the effect size for cognitive fusion was larger for the pre-treatment to follow-up period than for the pre- to post-treatment period. While the reason for this is not immediately certain, it may be that cognitive defusion is a skill that requires a longer time frame for practice and integration.

As predicted, zero-order correlations indicated that change in each psychological flexibility process variable was significantly correlated with change in physical and social functioning and depression in the expected direction during both assessment periods. In the regression analyses, change in psychological flexibility processes cumulatively explained 6–27 % of variance in the changes in functioning and depression over both time periods, even after controlling for changes in pain intensity. Consistent with previous findings (Vowles and McCracken 2008; McCracken et al. 2015; Scott and McCracken 2015), change in pain acceptance uniquely contributed to changes in treatment outcomes in six of the eight regression analyses. This is the first study to show that changes in cognitive fusion and committed action are related to changes in important treatment outcomes in chronic pain. Changes in cognitive fusion and committed action uniquely contributed to changes in treatment outcomes in four and three of the eight regression analyses, respectively.

The unique associations between change in individual processes and change in treatment outcomes appeared to depend, at least in part, on the assessment interval under examination. In the pre- to post-treatment interval, change in pain acceptance uniquely predicted each of the four outcomes. However, change in acceptance only predicted two outcomes in the follow-up interval. Change in committed action uniquely predicted change in depression in the pre- to post-treatment period, and both physical functioning and depression during the follow-up. This is consistent with the findings of a previous study showing that acceptance dominated in the prediction of post-treatment outcomes, whereas values-based action dominated in predicting follow-up outcomes (Vowles and McCracken, 2008). The relative time course of changes in the specific processes of psychological flexibility studied here is an important question for future investigation and treatment implementation. Future research could utilize more frequent assessments of these processes, such as weekly diary ratings (Vowles et al. 2014a), to more sensitively examine this question.

Relative to pain acceptance, the small magnitude of changes in cognitive fusion, decentering, and committed action may have limited their capacity to contribute uniquely to changes in treatment outcomes. Additionally, the moderate correlations among the psychological flexibility process measures used in this study may restrict our ability to determine the unique contribution of separate processes to treatment outcomes. Previous research suggests that separate measures of different facets of psychological flexibility may reflect both a general underlying construct *and* partially distinct components related to the processes under investigation here (Scott et al. 2015). Thus, the inter-correlations among the process measures in this study indicate that current assessment measures of these processes are not tapping entirely distinct constructs. This could reflect greater overlap in these constructs than originally proposed by the psychological flexibility model or poor performance in the ability of these measures to assess distinct aspects of these processes. To facilitate future measurement of these processes and investigation of treatment mechanisms, further refinement of these measures may be needed, for example, by limiting item content overlap, to maximize their discriminant validity (Scott et al. 2015).

An important avenue for future research and for developing clinical practice will be to determine how to maximize treatment changes in each of the processes of psychological flexibility. The effect sizes found in the current research are not as large as those found in some previous studies (McCracken and Gutiérrez-Martínez 2011; Wicksell et al. 2013). In the current study, pain acceptance showed the largest improvements, which is perhaps unsurprising considering that this process variable has received the most research and clinical attention. The results here suggest that treatment methods may need to be further developed to enhance their impact on cognitive fusion, decentering, and committed action.

The results of this study should be considered in light of several limitations. First, no control group was included and, therefore, definitive conclusions about the impact of the ACT intervention on the process and outcome variables cannot be made. Certain outcome variables, such as depression, may show a degree of spontaneous remission (Posternak and Miller 2001). However, it must be noted that the sample here included people with highly complex and longstanding chronic pain, including high levels of pain-related distress and disability. Previous data from a comparable sample showed a lack of significant improvement on measures of depression and disability while people were waiting (mean duration approximately 4 months) for a similar ACT-based interdisciplinary treatment for chronic pain (McCracken et al. 2005). These data suggest that the magnitude of change observed in the current sample of people with chronic pain is not simply due to naturally occurring fluctuations in these variables. The correlational design of the study also precludes causal statements about whether changes in psychological flexibility process variables preceded changes in functioning. Therefore, a randomized-controlled trial will be needed to determine the causal impact of the treatment on the outcome and process variables under study here, and to more rigorously test the mediating role of these processes in ACT treatment outcomes for people with chronic pain.

A large number of participants did not complete follow-up measures. Those who did not provide follow-up data scored significantly worse at post-treatment in terms of depression and social functioning and showed lower psychological flexibility as indicated by their scores on the measures of acceptance, fusion, and decentering. However, the magnitude of the differences in scores on these variables was small and, thus, the clinical significance of these differences remains unclear.

Another limitation is that the data were all collected using self-report questionnaires and, therefore, shared method variance may have partly accounted the associations among variables. Also, several items from the PHQ-9 are somatic in nature, and may thus overlap with participants’ reports of pain. Future research using multiple assessment methods, including measures of overt behavior patterns that do not rely exclusively on self-report, would be beneficial. Despite demonstrating good reliability in a number of previous studies in people with pain, the social functioning subscale showed poor reliability in the present study and, therefore, the replicability of the results from analyses using this subscale must be determined. Finally, there are indications that the SF-36 may lack sensitivity to detect change, which may have limited our ability to detect changes associated with treatment in this study (Busija et al. 2008).

Despite limitations, the current study adds to previous research examining the associations between psychological flexibility variables and patient functioning following ACT for chronic pain. The initial prediction that each of the psychological flexibility processes would change and that these changes would uniquely and significantly relate to changes in outcome was only partially supported. Nonetheless, evidence here suggests that changes in some of the processes of psychological flexibility may be linked to improvements in patient functioning, as predicted by the psychological flexibility model. Further research is needed to maximize the effectiveness of ACT for chronic pain, and to determine whether larger improvements in the processes of psychological flexibility under study here are associated with better patient outcomes, as predicted by the psychological flexibility model.
